# The Power of Good: A Leader's Personal Power as a Mediator of the Ethical Leadership-Follower Outcomes Link

**DOI:** 10.3389/fpsyg.2018.01094

**Published:** 2018-07-02

**Authors:** Daniela K. Haller, Peter Fischer, Dieter Frey

**Affiliations:** ^1^Chair of Social Psychology, Department of Psychology and Pedagogics Ludwig Maximilian University Munich, Munich, Germany; ^2^Chair of Social, Work, Organizational and Business Psychology, Department of Psychology, Pedagogics, and Sport Science Universität Regensburg, Regensburg, Germany

**Keywords:** ethical leadership, power, moral identity, commitment, work engagement, job satisfaction, leader effectiveness, extra effort

## Abstract

The study's goal was to examine the socially responsible power use in the context of ethical leadership as an explanatory mechanism of the ethical leadership-follower outcomes link. Drawing on the attachment theory (Bowlby, 1969/1982), we explored a power-based process model, which assumes that a leader's personal power is an intervening variable in the relationship between ethical leadership and follower outcomes, while incorporating the moderating role of followers' moral identity in this transformation process. The results of a two-wave field study (*N* = 235) that surveyed employees and a scenario experiment (*N* = 169) fully supported the proposed (moderated) mediation models, as personal power mediated the positive relationship between ethical leadership and a broad range of tested follower outcomes (i.e., leader effectiveness, follower extra effort, organizational commitment, job satisfaction, and work engagement), as well as the interactive effects of ethical leadership and follower moral identity on these follower outcomes. Theoretical and practical implications are discussed.

## Introduction

Baltasar Gracian, an ancient philosophical writer once said, “The sole advantage of power is that you can do more good”, recognizing that the socially responsible use of power leads to beneficial outcomes. As power use represents an essential element of leadership (Clements and Washbush, 1999), a decisive question arises about whether leaders in their function as power holders use their power to serve the greater good or abuse it for selfish ends. Moral scandals from top managers of global companies (Colvin, 2003) have highlighted the significance of power holders' ethical behavior for economic success, prompting both practitioners and academics to focus on the ethical dimension of leadership (Brown et al., 2005). These societal developments resulted in the evolvement of ethical leadership as a new leadership style, which has gained increasing scholarly interest (Brown et al., 2005; Brown and Treviño, 2006). Accordingly, empirical research has extensively demonstrated that ethical leadership is related to beneficial follower outcomes such as higher employee job satisfaction, performance, and organizational commitment (Treviño and Brown, 2014). Rooted in theories of social learning and social exchange (Brown and Treviño, 2006), a growing number of studies has begun to elucidate the empirically confirmed relationship between ethical leadership and follower outcomes by investigating diverse explanatory mechanisms (e.g., Piccolo et al., 2010). Extending this current state of research, we build on the conceptualization of leadership as an influential process through which followers form values, attitudes, and behaviors (Khuntia and Suar, 2004), and examine the role of power in the ethical leadership-outcome link for the first time.

**Figure 1:**
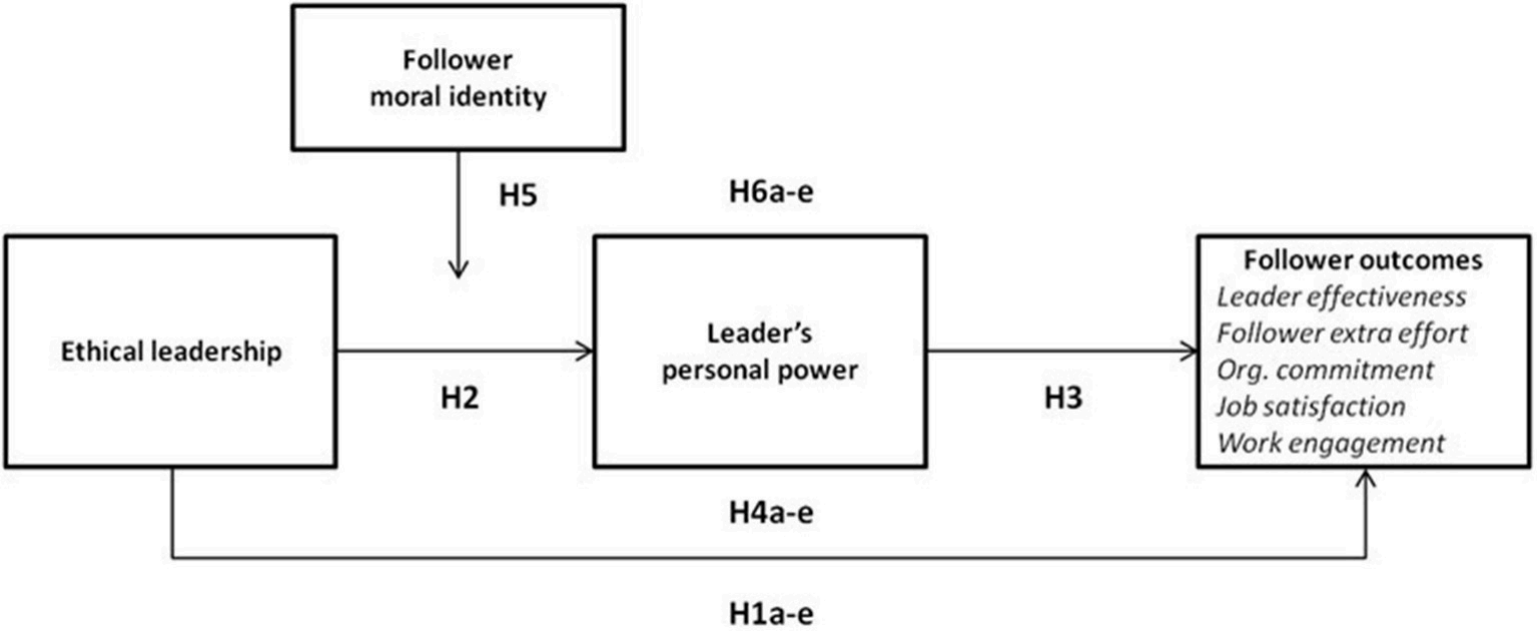
Hypothesized model of processes linking ethical leadership and follower outcomes, moderated by a follower's moral identity and mediated by the attribution of personal power bases to a leader.

By drawing on the attachment theory (Bowlby, 1969/1982) and integrating research on ethical leadership (Brown et al., 2005) and power bases (French and Raven, 1959), we propose a moderated mediation model, which captures the influencing process of ethical leadership on various follower outcomes from a power perspective (see Figure 1). We conceptualize ethical leadership as socially responsible power use (De Hoogh and Den Hartog, 2009), which involves with strong relational attachments (Bowlby, 1969/1982; Neubert et al., 2009). Thus, we examine a process model, which assumes that the attribution of personal power bases to a leader is a possible explanatory mechanism for the empirically substantiated relationship between ethical leadership and advantageous follower outcomes.

Consistent with current research on the role of a follower's personality in relation to the effects of ethical leadership (e.g., Avey et al., 2011; van Gils et al., 2015), we examine the moderating role of an employee's moral identity (Aquino and Reed, 2002) to elucidate how ethical leadership is related to follower outcomes. Empirical evidence suggests that the dispositional inclination to focus on morals (van Gils et al., 2015) shapes the relationship between ethical leadership and follower outcomes. Building on these findings, we examine the moderating function of moral identity in the relationship between ethical leadership and the attribution of personal power bases as well as the mediation of the interactive effects of ethical leadership and follower moral identity by personal power on follower outcomes.

Thus, we aim to extend the current research on ethical leadership in three ways. Building on theoretical considerations (De Hoogh and Den Hartog, 2009) and the attachment theory (Bowlby, 1969/1982), we first apply a power perspective on ethical leadership, empirically examining the effect of ethical leadership on a leader's personal power bases and integrating the concept of power with ethical leadership for the first time. This procedure ought to add a new defining element to the conceptualization of ethical leadership by explicating power use within the framework of ethical leadership.

Second, we address the call of van Knippenberg and Sitkin (2013) for enhanced exploration of leadership as a process and extend research on the mechanism of the ethical leadership-follower outcomes link. Thus, we examine a power-based psychological process that transfers ethical leadership into follower outcomes, adding new insight into ethical leadership's mechanism of action.

Finally, we follow the call for a deepened understanding of the employees' active role in the ethical leadership process (Den Hartog, 2015). Thus, we explore the moderating role of a self-concept-based personality variable for the first time—namely moral identity—in the relationship between ethical leadership and follower outcomes.

To test the proposed process model, we conduct a field-study (study 1) surveying employees at two measurement times and a scenario experiment (study 2) in which ethical leadership was experimentally manipulated as a dependent variable. Combining different methodologies, this procedure ensures a comprehensive examination of the hypothesized model, establishing a profound basis of conclusions.

## Theoretical background and hypotheses development

### Ethical leadership and follower outcomes

At all times, the ethical dimension of leadership has been regarded as particularly significant (Ciulla, 1998). Definitions of traditional scholarly work on ethical leadership are derived from a philosophical perspective, accentuating a prescriptive theoretical approach by establishing behavioral norms and moral standards that a leader should ideally meet (e.g., Kanungo and Mendonca, 1996). Since the year 2000, a descriptive approach has evolved from the emerging field of behavioral ethics, capturing ethical leadership as a social scientific construct by focusing on empirical data and validating the construct in the field (Treviño et al., 2000, 2003). Accordingly, construct development work was initiated with qualitative, interview-based field investigations within organizations by surveying corporate executives to characterize the behavior of executive-level ethical leaders (Treviño et al., 2000, 2003). Brown et al. (2005) synthesized findings from the field in the following formal constitutive definition of ethical leadership behavior, determining it as “the demonstration of normatively appropriate conduct through personal actions and interpersonal relationships, and the promotion of such conduct to followers through two-way communication, reinforcement, and decision-making” (p. 120). Thus, ethical leadership implies two distinct behavior levels: the “moral person” aspect, referring to distinct personality characteristics such as trustworthiness, honesty, and integrity, and the “moral manager” facet, meaning that an ethical leader explicitly focuses on ethics in his or her work behavior and proactively influences followers' ethical conduct by communicating the importance of ethics to followers and rewarding ethical behavior (Brown and Treviño, 2006).

Although Brown et al. (2005) regard ethical leadership as a one-dimensional construct, there is growing belief in the conceptualization of ethical leadership as a multidimensional construct (Resick et al., 2006; De Hoogh and Den Hartog, 2008; Kalshoven et al., 2011), criticizing the level of differentiation of ethical leadership behavior in the context of a one-dimensional concept (e.g., Eisenbeiss, 2012; Den Hartog, 2015). Consistently, research describes different leader behaviors as essentials of ethical leadership (see Den Hartog, 2015). For example, a cross-cultural study by Resick et al. (2006) states components of ethical leadership, such as character/integrity, ethical awareness, community/people orientation, motivating, encouraging, and empowering, as well as managing ethical accountability. Similarly, Kalshoven et al. (2011) developed the multidimensional ethical leadership at work (ELW) scale, which measures seven different dimensions of ethical leadership behavior, namely integrity, fairness, people orientation, power sharing, concern for sustainability, role clarification, and ethical guidance. Thus, “ethical leadership forms an overarching construct composed of multiple distinct, yet related, leader behaviors” (Den Hartog, 2015, p. 413). In this context, the scientific question arises whether the ethical leadership concept is culturally invariant. The current state of research indicates that the cross-cultural perceptions of characteristic ethical leader behaviors are similar to each other, though the specific significance of the single components of ethical leadership differs between cultures (Resick et al., 2006; Den Hartog, 2015).

The described critical scientific discussion about the theoretical concept of ethical leadership (Eisenbeiss, 2012) also raises an issue concerning conceptual differences of ethical leadership to other value-based leadership styles, such as transformational leadership (Bass, 1985). Transformational leadership and ethical leadership share essentials, such as the concern for others, ethical decision making, integrity, and role modeling; these typical transformational leadership behaviors are anchored in the moral person dimension of ethical leadership (Brown et al., 2005; Brown and Treviño, 2006). However, there are substantial differences, which are also reflected by the incremental validity of ethical leadership in predicting outcomes (Brown et al., 2005; Brown and Treviño, 2006), though more profound research on the discriminant validity of the ethical leadership concept is needed (Den Hartog, 2015). Thus, transformational leadership focus on motivating followers by an inspiring vision and offering intellectual stimulation and can also be implemented in an unethical way (i.e., pseudo transformational leadership; Bass and Steidlmeier, 1999, see Brown and Treviño, 2006; Den Hartog, 2015). Conversely, ethical leadership explicitly focus on the ethical aspects of leadership and includes transactional behaviors, such as emphasizing ethical standards and reinforcing followers' ethical conduct, which is rooted in the moral manager dimension of ethical leadership (Brown and Treviño, 2006).

Combining transformational and transactional elements, ethical leadership is characterized by high efficiency and management success because the most effective leadership style is both transformational and transactional (Waldman et al., 1990). An enormous amount of research on outcomes of ethical leadership consistently provides evidence for the beneficial impact of ethical leadership (Treviño and Brown, 2014). In addition to organizational citizenship behavior (e.g., Avey et al., 2011; DeConinck, 2015), and employee (Piccolo et al., 2010; Walumbwa et al., 2011; Bouckenooghe et al., 2015) and firm performance (e.g., Eisenbeiss et al., 2015), ethical leadership is positively related to leader effectiveness (Brown et al., 2005; De Hoogh and Den Hartog, 2008; Toor and Ofori, 2009; Kalshoven et al., 2011). Moreover, ethical leadership is associated with advantageous job attitudes and job-related affective states, such as trust (Den Hartog and De Hoogh, 2009; Kalshoven et al., 2011), an employee's willingness to put in extra effort (Brown et al., 2005; Toor and Ofori, 2009; Eisenbeiss and van Knippenberg, 2015), organizational commitment (Den Hartog and De Hoogh, 2009; Neubert et al., 2009; Hassan et al., 2013; Demirtas and Akdogan, 2015), job satisfaction (Neubert et al., 2009; Kalshoven et al., 2011), and work engagement (Chughtai et al., 2015; Demirtas et al., 2015), while reducing employee deviance (Mayer et al., 2010; Avey et al., 2011; van Gils et al., 2015) and turnover intentions (DeConinck, 2015; Demirtas and Akdogan, 2015).

To enhance the validity and the scope of our proposed power-based process model of the ethical leadership-follower outcomes-link, we aim at testing a broad range of distinct follower outcomes which have empirically substantiated relations to ethical leadership. Thus, we focus on leader effectiveness as a leader's performance indicator in the context of our studies and on the following four different beneficial follower job attitudes: follower extra effort, organizational commitment, job satisfaction, and work engagement. Generally defined in terms of the ability to attain goals (Bass, 2008), leader effectiveness captures a leader's performance as perceived by his or her followers (Felfe, 2006). Follower extra effort implies a dedicated effort on the job (Campbell, 1990), including behavior that exceeds common role expectations (Seltzer and Bass, 1990), mirrored by “the willingness (…) to exert additional time and energy to achieve organizational goals” (Webb, 2007, p. 58). Alternatively, job satisfaction reflects an emotional response to a job as whole or specific aspects of a job resulting from a cognitive process of comparing real circumstances with individual expectations (Smith et al., 1969; Locke, 1976).

Organizational commitment captures the bond strength between an organization and an employee, which consists of three components: affective, normative, and continuous commitment (Meyer and Allen, 1991). Affective commitment describes an “emotional attachment to, identification with, and involvement in the organization” (Meyer et al., 2002, p. 21), and normative commitment captures the felt obligation to remain in an organization, whereas continuous commitment reflects the perceived necessity to stay due to the anticipated costs of leaving (Meyer and Allen, 1991). In contrast to continuous commitment, affective and normative commitments are deemed positive and beneficial forms of commitment due to their consequences regarding an employee's behavior and state of mind (Meyer et al., 2002). Ethical leadership is consistently positively associated with affective and normative commitment, while exhibiting a negative relation to continuous commitment (see Den Hartog and De Hoogh, 2009 for a further discussion). Thus, the conceptualization of organizational commitment in this study refers to affective and normative commitment.

Empirically distinct from organizational commitment (Hallberg and Schaufeli, 2006), we explore a fifth outcome work engagement, which is an indicator of occupational wellbeing. Work engagement describes a “positive, fulfilling work-related state of mind that is characterized by vigor, dedication, and absorption” (Schaufeli et al., 2002, p. 74). Vigor refers to a high amount of energy and mental resilience at work, and an investment of effort and persistence when considering obstacles. Dedication implies strong work involvement, coinciding with a sense of significance and feelings of enthusiasm, pride, and inspiration. Absorption is characterized by being fully immersed in one's work tasks, accompanied with losing a sense of time and difficulties detaching from work (Schaufeli et al., 2002). On the solid basis of current empirical research, we propose:

H1a: ethical leadership is positively related to leader effectiveness.
H1b: ethical leadership is positively related to follower extra effort.
H1c: ethical leadership is positively related to organizational commitment.
H1d: ethical leadership is positively related to job satisfaction.
H1e: ethical leadership is positively related to work engagement.

### The mediating role of personal power in the ethical leadership-follower outcomes link

The robust evidence on the beneficial effects of ethical leadership on follower outcomes elicits questions relating to the explanatory mechanism of this correlation. The common theoretical explanations for the relationship between ethical leadership and follower outcomes are rooted in the social learning theory (Bandura, 1986) and social-exchange theory (Blau, 1964; see Brown and Treviño, 2006). The framework of the social learning theory suggests that ethical leaders influence their employees' conduct by role modeling (Brown et al., 2005). Thus, followers imitate appropriate behavior by observing ethical leaders, who represent attractive and credible role models for the impartation of decent and prosocial behavior (Brown and Treviño, 2006). Similarly, the social exchange theory perspective on the relationship between ethical leadership and positive follower outcomes implies that followers of ethical leaders tend to consider themselves in a social exchange relationship with their leader, encouraging the development of trust and the evolvement of reciprocity norms in the leader-follower relationship, which results in beneficial follower outcomes (Brown and Treviño, 2006).

Based on these theoretical considerations, several empirical studies examined diverse mediating mechanisms in the relationship between ethical leadership and follower outcomes. Thus, empirical evidence suggests the mediating role of environment factors, such as ethical climate (Neubert et al., 2009; Demirtas and Akdogan, 2015), task-related factors such as meaningfulness of work (Piccolo et al., 2010; Demirtas et al., 2015), employees' internal states such as self-efficacy (e.g., Walumbwa et al., 2011) or psychological capital (Bouckenooghe et al., 2015), and mediating mechanisms associated with the leader-follower-relationship such as leader-member exchange (Walumbwa et al., 2011; Hassan et al., 2013) and trust (Chughtai et al., 2015; Mo and Shi, 2017).

Although the described studies indicated several mediating mechanisms that explain the influence of ethical leadership on follower outcomes, the influence process of the ethical leadership-follower outcomes link has not been examined from a power perspective. Leadership as an influential process through which followers form values, attitudes, and behaviors (Khuntia and Suar, 2004) is implicitly interrelated with power (Northouse, 2007; Bass, 2008), which is defined as the potency to influence (French and Snyder, 1959; Janda, 1960). Accordingly, French and Raven (1959) define five bases of power, indicating different forms in which power can be used by leaders to influence followers' behavior and outcomes. Legitimate, coercive, and reward power are classified as positional power bases since they derive solely from the occupation of a position in an organizational system (Yukl and Falbe, 1991; Northouse, 2007; Bass, 2008). Thus, legitimate power describes the formal authority of a position, while reward and coercive power represent the perceived potency to grant benefits or disadvantages to followers (French and Raven, 1959; Hinkin and Schriesheim, 1989).

By contrast, personal power comprises expert and referent power, stemming from a leader's personal attributes and appearance, and thus representing incremental potency to influence (Student, 1968; Rahim, 2009). Manifesting as an emotional bond between leader and follower, personal power enables a leader to strengthen relationships with others by conveying affiliation, respect, and appreciation (Northouse, 2007; Bass, 2008). Expert power involves the capacity to grant information, knowledge, and expertise. This power base is reflected by job-related skills, accurate decisions, correct perception of reality, problem-solving competence, as well as a rational and reliable judgment by the leader, resulting in the perception of competence on the part of the employees (French and Raven, 1959; Hinkin and Schriesheim, 1989). Referent power describes the ability to convey feelings of personal acceptance and respect to subordinates, and it is based on followers' identification with and attraction to their leader, as indicated by followers' admiration and respect for a leader and by perceiving him or her as a role model (French and Raven, 1959).

Research shows that followers' perceptions of a leader's power bases depend on leadership behavior since behavioral cues convey power messages (e.g., Gioia and Sims, 1983; Hinkin and Schriesheim, 1990). Accordingly, findings demonstrate that followers' perceptions of their leader's power bases are related to distinct leadership styles (Ansari, 1990; Atwater and Yammarino, 1996; Barbuto et al., 2001; Pierro et al., 2013). For example, a positive relationship between transformational leadership and personal power is empirically confirmed (Atwater and Yammarino, 1996; Pierro et al., 2013). Thus, leadership behavior affects followers' perceptions of a leader's social power. We assume that also ethical leadership behavior is related to attributing specific corresponding power bases to a leader. In this context, the question arises, which form of power use is characteristic of an ethical leader.

Representing a key element of the relationship between a supervisor and his or her subordinates (Yukl, 2006), power should be used by leaders to promote collective goals since the prevalent definition of power as the potency to control others' outcomes and resources (e.g., Fiske, 1993) implicitly links power with a facet of morality, namely the concern and responsibility for the welfare of others (Keltner et al., 2006). This link between social responsibility and power is manifested within the conceptualization of ethical leadership. From the perspective of social influence and power, the socially responsible use of power is a key element of ethical leadership (De Hoogh and Den Hartog, 2009). In this sense, ethical leadership is defined as “the process of influencing in a socially responsible way the activities of an organized group toward goal achievement” (De Hoogh and Den Hartog, 2009, p. 341). This definition implies an explicit emphasis on the means through which an ethical leader aims to achieve individual and collective goals, extending the general definition of leadership by Stogdill (1950 see De Hoogh and Den Hartog, 2009). Consequently, ethical leadership as a specific form of power use should be associated with followers' perceptions of distinct corresponding power bases^1^.

Building on De Hoogh and Den Hartog (2009) theoretical arguments and additionally drawing on the attachment theory (Bowlby, 1969/1982), we argue that ethical leadership is related to the attribution of personal power to a leader (see also Neubert et al., 2009).

The attachment theory originally describes the child-parent relationship, in which the child represents the needy and dependent relationship partner, whereas the parent has the role of the stronger and wiser caregiver or attachment figure (Bowlby, 1969/1982; Davidovitz et al., 2007). The resulting relational attachments can be defined as emotional bonds that are built, as one relationship partner meets the needs of another (Bowlby, 1969/1982). However, the leader-follower relationship can also be captured in terms of relational attachments, since these relationship partners interact in close proximity and the attachment figure (i.e., the leader) potentially offers support and security (Popper and Mayseless, 2003; Davidovitz et al., 2007). Core components of ethical leadership behavior consists of showing respect, protecting employees' interests and offering individually considerate support (Brown et al., 2005; Kalshoven et al., 2011), which leads to strong relational attachments (Davidovitz et al., 2007; Neubert et al., 2009). From a power perspective, this emotional bond between leader and follower manifests in the attribution of personal power to the leader by the follower (Northouse, 2007; Bass, 2008, see also Neubert et al., 2009), as leaders generally promote their personal power by showing respect and protecting their employees' interests (Bass, 2008). Thus, on the basis of strong relational attachments, ethical leadership behavior should enhance a leader's personal power. More precisely, ethical leadership behavior should be associated with expert power, which is reflected by fair decisions and objective judgment (French and Raven, 1959; Hinkin and Schriesheim, 1989), as ethical leaders make fair decisions, judge in an ethical manner, and clearly determine responsibilities, expectations, goals, and guidelines for ethical conduct (Brown et al., 2005; Kalshoven et al., 2011). Similarly, ethical leadership behavior should also be related to followers' perceptions of referent power, since acting as a role model and behaving respectfully, considerately, and in a caring manner - core behavioral characteristics of an ethical leader (Brown et al., 2005) - contributes significantly to a leader's referent power (Northouse, 2007). Hence, we propose:

H2: Ethical leadership is positively related to a leader's personal power.

Personal power bases are generally regarded as essentially more positive than positional power bases, as a very robust empirical picture indicates that the use of person-based power is the most effective (Podsakoff and Schriesheim, 1985; Carson et al., 1993; Yukl, 2006; Rahim, 2009). Thus, whereas the findings on the relationship between position-based power and followers' outcomes are mixed, indicating comparatively reduced effectiveness (Bachman et al., 1966; Podsakoff and Schriesheim, 1985; Yukl, 2006), personal power shows many positive relations with indicators of beneficial follower outcomes, such as performance (Student, 1968; Podsakoff and Schriesheim, 1985; Rahim et al., 1994), job satisfaction (Bachman et al., 1966; Rahim and Afza, 1993; Rahim et al., 1994), satisfaction with a supervisor (Bachman et al., 1966; Podsakoff and Schriesheim, 1985), and reduced turnover and absenteeism (Student, 1968; Podsakoff and Schriesheim, 1985; Rahim and Afza, 1993). Furthermore, personal power is positively associated with commitment and compliance since it leads to comparatively high personal involvement, explaining enhanced compliance and engagement that goes beyond what is necessary (Bachman et al., 1966; Yukl and Falbe, 1991; Rahim et al., 1994). Referring to the robust research status regarding the positive effect of personal power on follower outcomes, we hypothesize:

H3a: personal power is positively related to leader effectiveness.
H3b: personal power is positively related to follower extra effort. power is positively related to organizational commitment.
H3c: personal power is positively related to job satisfaction.
H3d: personal power is positively related to work engagement.

In summary, we propose that the perception of a leader's personal power bases plays a key role in the ethical leadership-follower outcomes link, mediating the positive relationship between ethical leadership and follower outcomes. On the basis of strong relational attachments, the specific pattern of ethical leadership behavior should enhance employees' perceptions of their leader's personal power bases. As personal power is characterized by high organizational effectiveness, it should in turn promote advantageous follower outcomes. Consequently, we propose:

H4a: personal power mediates the positive relationship between ethical leadership and leader effectiveness.
H4b: personal power mediates the positive relationship between ethical leadership and follower extra effort.
H4c: personal power mediates the positive relationship between ethical leadership and organizational commitment.
H4d: personal power mediates the positive relationship between ethical leadership and job satisfaction.
H4e: personal power mediates the positive relationship between ethical leadership and work engagement.

### The moderating role of an employee's moral identity

Followers' perceptions of leadership behavior are dependent on social information processing (Salancik and Pfeffer, 1978) and as a result, they are contingent on followers' own cognitive reference framework (e.g., Lord and Maher, 1991; van Quaquebeke et al., 2011). Therefore, individual differences influence the perception and evaluation of leadership behavior (Vecchio and Boatwright, 2002), resulting in diverging follower outcomes (e.g., Graen and Uhl-Bien, 1995; Gerstner and Day, 1997). Recent research indicates that the effects of ethical leadership on follower outcomes are not invariant, but are dependent on an employee's personality (Avey et al., 2011; Eisenbeiss and van Knippenberg, 2015; van Gils et al., 2015). Thus, personality variables which are characterized by a higher focus and perceived subjective importance on morality, such as moral attentiveness, moral emotions, and mindfulness, enhance the effects of ethical leadership on follower outcomes, for example follower helping or extra effort (Eisenbeiss and van Knippenberg, 2015; van Gils et al., 2015).

In alignment with this former research we aim to examine the moderating role of moral identity in the ethical leadership-follower outcome link, since empirical evidence indicates the significant function of an employee's self-concept regarding perceptions of leadership (e.g., Lord and Brown, 2004; Dinh et al., 2013). The role of a leader's moral identity with respect to the emergence of ethical leadership behavior has been confirmed (e.g., Mayer et al., 2012). Furthermore, several studies indicate that ethical leadership enhances an employee's moral identity (Wen and Chen, 2016; Gerpott et al., 2017; Bavik et al., 2018). However, the function of an employee's moral identity in the processing of ethical leadership behavior related to the evolution of follower outcomes has not been explored to date.

Moral identity is defined as an individual's organized associative cognitive network (schema) of moral virtues (e.g., being generous), feelings (e.g., concern for others), and behaviors (e.g., helping others). Within this schema, the strength of these moral associations mirrors the extent to which morality is part of one's self-concept (Aquino and Reed, 2002; Reed and Aquino, 2003). Thus, individuals' moral identities differ in their significance within a person's entire self-definition, influencing the processing of morality-related social information (Aquino and Reed, 2002; Reed and Aquino, 2003) and subsequent judgment (e.g., Reed et al., 2007). Accordingly, a study confirmed that employees' reactions to supervisor abuse are shaped by the employees' level of moral identity (Greenbaum et al., 2013). Similarly, findings confirm the moderating role of moral identity in processing ethical leadership behavior in relation to customer-related outcomes, such as purchasing intentions (van Quaquebeke et al., 2017; Wu, 2017). Thus, we propose that a follower's moral identity may also shape the relationship between ethical leadership and follower outcomes by moderating the proposed link between ethical leadership and personal power bases.

Empirical evidence indicates that personality generally influences the perception of power bases (Lord et al., 1980). Since ethical leadership is predominantly characterized by a leader's moral behavior, the level of a follower's moral identity might determine the amount of attributed personal power bases due to the chronically strong link between morals and self-conception (Aquino and Reed, 2002). In this vein, the level of follower moral identity might define the amount of relational attachments, which result from ethical leadership behavior and are reflected by the attribution of personal power (Bowlby, 1969/1982; Bass, 2008).

The attribution of referent power is mainly dependent on perceiving the leader as a role model and feeling sympathy and appreciation for him or her (French and Raven, 1959). Thus, an employee, who perceives a higher importance of moral behavior due to his or her highly developed moral identity, may attribute more referent power to an ethical leader compared to an employee with a rather low moral identity. Similarly, the attribution of expert power is contingent on the perception of a leader's decision making, objective judgment, and competence (French and Raven, 1959; Hinkin and Schriesheim, 1989). As outlined above, in case of ethical leadership, fair decision making, ethical judgment, and establishing and forcing ethical guidelines may contribute to the attribution of expert power, reflecting a dependence on the ethical content of leadership behavior. Thus, an employee with a high moral identity may attribute more expert power to an ethical leader than a follower with a moderately developed moral identity, since they differ in the “centrality of morality to self” (Aquino and Reed, 2002, p. 1424) and in the subjectively perceived significance of the moral aspects of a leader's behavior. Hence, we propose:

H5: an employee's moral identity moderates the relationship between ethical leadership and the attribution of personal power to a leader, such that ethical leadership has a stronger positive impact on the attribution of personal power to a leader for employees with a higher moral identity as compared to those with a lower moral identity.

Based on the discussion above, we finally argue that personal power also mediates the interactive effect of ethical leadership and an employee's moral identity on diverse follower outcomes. Although differences in the manifestation of an employee's moral identity are associated with how an employee responds to ethical leadership behavior, attributing diverse corresponding levels of personal power to his or her leader, the perceived amount of a leader's personal power should play an intervening key role in the relationship between ethical leadership and follower outcomes. Following the preceding discussion, we argue that in the case of an employee's highly developed moral identity, the effect of ethical leadership on personal power and ultimately on the various follower outcomes will be stronger than in the case of a rather low moral identity. Hence, we hypothesize:

H6a: personal power mediates the interactive effect of ethical leadership and an employee's moral identity on leader effectiveness.
H6b: personal power mediates the interactive effect of ethical leadership and an employee's moral identity on follower extra effort.
H6c: personal power mediates the interactive effect of ethical leadership and an employee's moral identity on organizational commitment.
H6d: personal power mediates the interactive effect of ethical leadership and an employee's moral identity on job satisfaction.
H6e: personal power mediates the interactive effect of ethical leadership and an employee's moral identity on work engagement.

## Study 1 (field study)

### Materials and methods

#### Sample and procedure

The field study was conducted online and in two phases. Internet recruitment methods are increasingly popular among researchers and their use has been approved by the American Psychological Association's Board of Scientific Affairs' Advisory Group (Kraut et al., 2004). Thus, participants were recruited via postings on university-related and professional-network social media platforms. The incentive comprised the opportunity to participate in a lottery; being in an employment relationship (full- or part-time) defined the requirement for participation. Since data were collected in two waves, participants could voluntarily sign up for the second survey, to which they were automatically invited via E-mail 2 weeks after completing the first survey. The separate questionnaires were matched on basis of a code, ensuring anonymity and confidentiality. This form of data collection follows established methodological recommendations, as the common method variance in single-source data is significantly minimized by temporally separating the data collection of the independent and dependent variables (Podsakoff et al., 2003, 2012). Thus, in phase 1, employees assessed their leader's ethical leadership behavior, rated their own moral identity, and provided information about control variables and demographic data. In phase 2, the participants reported the attribution of personal power bases to their leader and follower outcomes (i.e., leader effectiveness, follower extra effort, job satisfaction, organizational commitment, and work engagement).

During the survey period of about 6 weeks, 251 employees completed the first part of the field study and 235 completed the second part, corresponding to a response rate of 93.6%.

65.5% of the final sample was female, with an average age of 30.1 years (*SD* = 7.6) and a rather high educational level (4.7% secondary school leaving certificate; 16.2% higher education entrance; 79.2% university degree). Furthermore, 62.1% of the employees worked full-time, stemming from a wide range of branches (business & finance sectors: 14.0%; services & trade sectors: 20.4%; health & social sectors: 11.5%; education & consulting sectors: 7.7%; industry & craft sectors: 12.3%; science sector: 9.4%; public services: 8.5%; gastronomy: 1.7%; others: 14.5%).

#### Measures

Since this study was conducted in Germany, every English scale that was not available in a German version was translated following the standard procedure of translation and independent back-translation (Brislin, 1980) by employing independent qualified translators. This procedure is consistent with the international test commission guidelines for translating and adapting tests (International Test Commission, 2017), considering the given, very similar cultural contexts (i.e., western countries; see Van de Vijver and Hambleton, 1996). Participants rated all measures on seven-point Likert-scales ranging from one (strongly disagree) to seven (strongly agree).

##### Ethical leadership

Employees assessed ethical leadership with the validated German Version of the ten-item ELS scale (Rowold et al., 2009), originally developed by Brown et al. (2005). Sample items include: “My leader listens to what employees have to say” and “My leader sets an example of how to do things the right way in terms of ethics” (α = 0.90).

##### Personal power bases

Employees' perceptions of their leader's personal power bases were measured by the corresponding two subscales, expert power and referent power, from the original French and Raven (1959) five power bases measure by Hinkin and Schriesheim (1989). Each personal power base was reported by four items, such as “My supervisor can provide me with sound job-related advice” (expert power) and “My supervisor can make me feel important” (referent power). As the combination of expert power and referent power into the higher order construct personal power is both theoretically substantiated and empirically supported by diverse factor structure tests (e.g., Student, 1968; Yukl and Falbe, 1991; Peiró and Meliá, 2003; Northouse, 2007; Bass, 2008), the two subscales were merged into one score for personal power (α = 0.94).

##### Moral identity

Participants reported their own moral identity by Aquino and Reed's five-item subscale internalization (α = 0.67), capturing the degree to which a person's moral identity is core to his or her sense of self (Aquino and Reed, 2002). The introduction followed the recommendations of Aquino and Reed (2002); one sample item is “It would make me feel good to be a person who has these characteristics.” As internal consistence is also dependent on the number of items and the sample size and the scale is quite short, a cronbach's alpha of α = 0.67 can be considered as acceptable (Churchill and Peter, 1984; Loewenthal, 2004).

##### Leader effectivenesss

Perceived leader effectiveness was measured by a four-item scale from the German validated version (Felfe, 2006) of the Multi-Leadership Questionnaire (MLQ; Bass and Avolio, 1995). One sample item is “My leader is effective in meeting organizational requirements” (α = 0.86).

##### Follower extra effort

A leader's capacity to elicit extra effort from an employee was assessed using the three-item scale of the German validated version (Felfe, 2006) of the MLQ (Bass and Avolio, 1995), including items such as “My leader gets me to do more than I expected to do” (α = 0.94).

##### Organizational commitment

Organizational commitment (α = 0.88) was measured by the two subscales of affective commitment and normative commitment, developed by Meyer et al. (1993). The 12-item measure includes items, such as “I would be very happy to spend the rest of my career with this organization” (affective commitment) and “I owe a great deal to my organization” (normative commitment).

##### Job satisfaction

The participants reported their job satisfaction based on the five-item subscale global job satisfaction from the shortened German adaption “KAFA” (Haarhaus, 2016) of the job-descriptive index (Smith et al., 1969). One sample item is “In total, my job is satisfactory” (α = 0.90).

##### Work engagement

We measured work engagement with the nine-item version of the Utrecht Work Engagement Scale (UWES-9) developed by Schaufeli et al. (2006), capturing three facets of work engagement with three items each (α = 0.94). Exemplary items include “At my job, I feel strong and vigorous” (vigor), “My job inspires me” (dedication), and “I feel happy when I am working intensely” (absorption).

#### Control variables

In addition to the form of occupation (1 = “full-time,” 2 = “part-time”), we controlled for tenure with leader (1 = < 6 months to 5 > 5 years), employee educational level (1 = no school-leaving qualification to 7 = doctoral degree), and leader and employee sex (1 = “male,” 2 = “female”). Controlling for leader tenure resulted from empirical evidence indicating the strong influence of leader tenure on perceptions of leadership behavior (e.g., Wayne et al., 1997). The control for an employee's education level was based on findings suggesting that education is an important determinant of moral competence and moral judgment (e.g., Lind, 1993). Furthermore, we controlled for employee sex due to sex differences in moral judgment (e.g., Wark and Krebs, 1996), and for leader's sex due to the male tendency to act less ethically (e.g., Swamy et al., 2001) and the influence of a leader's sex on followers' perceptions of his or her ethics (Schminke et al., 2003).

#### Construct validity

Following the recommendations of Brown (2006), we conducted a series of confirmatory factor analyses with the aid of AMOS to test the discriminant validity of the single-source study variables, referring to chi-square statistics and fit indices of RMSEA, IFI, and CFI (Anderson and Gerbing, 1988; Joreskog, 1993). To meet sample size guidelines for parameter estimation (e.g., Landis et al., 2000) and enhance indicator stability (e.g., West et al., 1995), we used the subscales for the constructs of personal power, work engagement, and organizational commitment. The hypothesized 8-factor model of ethical leadership, personal power, moral identity, leader effectiveness, follower extra effort, organizational commitment, job satisfaction, and work engagement, χ^2^ (413, *N* = 235) = 805.97, *p* < 0.001; RSMEA = 0.06; IFI = 0.92 and CFI = 0.92, yielded a better fit to the data than a one-factor model—where all indicators were set to load on a single factor, χ^2^ (527, *N* = 235) = 2949.40, *p* < 0.001; RSMEA = 0.14; IFI = 0.57 and CFI = 0.56, supporting the distinctiveness of the eight study variables for subsequent analyses.

### Results

Table 1 implies descriptive statistics and bivariate correlations.

**Table 1:** Descriptive statistics and correlations.

	**Variable**	***M***	***SD***	**1**	**2**	**3**	**4**	**5**	**6**	**7**	**8**	**9**	**10**	**11**	**12**	**13**
1	Ethical leadership	4.58	1.14	1												
2	Moral identity	5.93	0.82	0.12	1											
3	Personal power	5.26	1.27	0.64^***^	0.14^*^	1										
4	Leader effectiveness	4.86	1.28	0.65^***^	0.09	0.81^***^	1									
5	Follower extra effort	4.47	1.51	0.50^***^	0.17^**^	0.78^***^	0.72^***^	1								
6	Org. commitment	3.82	1.11	0.43^***^	0.08	0.42^***^	0.46^**^	0.45^***^	1							
7	Job satisfaction	5.34	1.20	0.52^***^	0.11	0.56^***^	0.52^***^	0.48^***^	0.62^***^	1						
8	Work engagement	4.43	1.24	0.40^***^	0.05	0.46^***^	0.44^***^	0.54^***^	0.54^***^	0.67^***^	1					
9	Employee sex	1.66	0.48	0.02	0.18^**^	0.02	−0.04	0.02	−0.01	−0.04	−0.03	1				
10	Employee education	5.34	0.96	0.10	0.12	0.06	0.09	0.09	0.09	0.18^**^	−0.19^***^	−0.01	1			
11	Leader sex	1.31	0.46	−0.13^*^	−0.03	−0.17^*^	−0.19^**^	−0.09	−0.12	−0.17^**^	−0.13	0.21^***^	.02	1		
12	Leader tenure	2.38	1.23	−0.15^*^	−0.05	−0.05	−0.08	−0.07	0.07	0.08	0.09	−0.02	0.07	−0.09	1	
13	Occupation form	1.38	0.49	−0.02	−0.04	0.07	0.00	0.03	−0.14^*^	−0.07	−0.11	0.11	−0.27^***^	0.11	0.00	1

N = 235. For employee and leader sex, 1 = male, 2 = female. For occupation form, 1 = full-time, 2 = part-time. Org.,organizational.

* p < 0.05;

** p < 0.01;

*** *p < 0.001*.

To test hypotheses H1a-e, we conducted ordinary least squares (OLS) regressions, predicting the diverse follower outcomes of ethical leadership. In each case, we controlled for employee's sex, employee's education, leader's sex, tenure with the leader, and occupation form. The results show that ethical leadership is positively related to leader effectiveness (*b* = 0.71, β = 0.63, *SE* = 0.06, *p* < 0.001), follower extra effort (*b* = 0.65, β = 0.49, *SE* = 0.08, *p* < 0.001), organizational commitment (*b* = 0.43, β = 0.44, *SE* = 0.06, *p* < 0.001), job satisfaction (*b* = 0.54, β = 0.51, *SE* = 0.06, *p* < 0.001), and work engagement (*b* = 0.44, β = 0.40, *SE* = 0.07, *p* < 0.001), substantiating hypotheses 1a-e.

Hypothesis testing regarding the mediation and moderated mediation models was based on path analytic procedures (Edwards and Lambert, 2007; Preacher et al., 2007) and bootstrapping analyses to assess the (conditional) indirect effects (Shrout and Bolger, 2002), using the SPSS macro PROCESS (Preacher et al., 2007; Hayes, 2013). As recommended by Hayes and Cai (2007), we used a heteroskedasticity-consistent standard error estimator for the OLS regressions to prevent biased confidence intervals and mean-centered variables used as a component in interaction terms to avoid multi-collinearity (Cohen et al., 2003).

The path analytic procedures consist of two steps (see Edwards and Lambert, 2007; Hayes, 2013). In the first step, the mediator variable (i.e., personal power) is regressed on the independent variables and their interaction term in case of moderated mediation (mediation model: ethical leadership, moderated mediation model: ethical leadership and moral identity). The results of the regression analyses for the first-stage dependent variable personal power are depicted in Table 2. The second step predicts the dependent variables (i.e., follower outcomes) from the mediator (personal power) and the predictor (ethical leadership), and the results of the second-stage dependent variables are shown in Table 3.

**Table 2:** First paths of the (moderated) mediation models, predicting the first stage dependent variable personal power.

**Models**	**Mediation**			**Moderated mediation**		
**Variable**	**Personal power**			**Personal power**		
	***b***	β	***SE***	***b***	β	***SE***
Ethical leadership	**0.71**	0.64^***^	**0.07**	0.66	0.59^***^	0.07
Moral identity				0.11	0.07	0.06
EL x moral identity				**0.25**	0.18^*^	**0.08**
Employee sex	0.05	0.02	0.05	0.00	0.00	0.06
Employee education	0.03	0.02	0.06	0.03	0.02	0.06
Leader sex	−0.26	−0.09	0.06	−0.25	−0.09	0.06
Leader tenure	0.03	0.03	0.05	0.02	0.02	0.04
Occupation form	0.27	0.10	0.05	0.24	0.09	0.05
*F*	19.03^***^			19.03^***^		
*R*^2^	0.43			0.47		

N = 235. For employee and leader sex, 1 = male, 2 = female. For occupation form, 1 = full-time, 2 = part-time. EL = Ethical leadership. Standard errors are based on standardized coefficients. Values in bold are relevant to hypothesis tests.

* p < 0.05;

**p < 0.01;

*** *p < 0.001*.

**Table 3:** Second paths of the (moderated) mediation models, predicting the second stage dependent variables, i.e., follower outcomes.

	**Leader effectiveness**			**Follower extra effort**			**Org. commitment**			**Job satisfaction**			**Work engagement**		
**Variable**	***b***	**β**	***SE***	***b***	**β**	***SE***	***b***	**β**	***SE***	***b***	**β**	***SE***	***B***	**β**	***SE***
Ethical leadership	0.23	0.20^***^	0.06	−0.02	−0.01	0.06	0.26	0.27^***^	0.08	0.29	0.27^***^	0.07	0.19	0.17^*^	0.07
Personal power	**0.68**	0.67^***^	**0.06**	**0.94**	0.79^***^	**0.06**	**0.23**	0.27^***^	**0.08**	**0.36**	0.38^***^	**0.08**	**0.35**	0.35^***^	**0.07**
Employee sex	−0.12	−0.05	0.04	−0.00	−0.00	0.04	0.01	0.00	0.06	−0.08	−0.03	0.05	−0.07	−0.03	0.06
Employee education	0.03	0.02	0.04	0.07	0.04	0.05	−0.00	−0.00	0.06	0.14	0.11^*^	0.05	0.15	0.12^*^	0.06
Leader sex	−0.11	−0.04	0.04	0.12	0.04	0.04	−0.02	−0.01	0.05	−0.13	−0.06	0.05	−0.05	−0.02	0.06
Leader tenure	−0.02	−0.02	0.04	−0.04	−0.03	0.05	0.11	0.12^*^	0.06	0.12	0.12^**^	0.04	0.13	0.12^*^	0.06
Occupation form	−0.07	−0.03	0.04	−0.06	−0.02	0.04	−0.35	−0.15^*^	0.06	−0.12	−0.05	0.05	−0.24	−0.09	0.06
*F*		63.20^***^			49.67^***^			11.37^***^			17.63^***^			713.24^***^
*R*^2^		0.69			0.61			0.26			0.39			0.28

N = 235. For employee and leader sex, 1 = male, 2 = female. For occupation form, 1 = full-time, 2 = part-time. Org., organizational. Standard errors are based on standardized coefficients. Values in bold are relevant to hypothesis tests.

* p < 0.05;

** p < 0.01;

*** *p < 0.001*.

The results from the mediation models indicate that ethical leadership was positively related to personal power (*b* = 0.71, β = 0.64, *SE* = 0.07, *p* < 0.001), confirming hypothesis 2. In addition, personal power was positively associated with each follower outcome, including leader effectiveness (*b* = 0.68, β = 0.67, *SE* = 0.06, *p* < 0.001), follower extra effort (*b* = 0.94, β = 0.79, *SE* = 0.06, *p* < 0.001), organizational commitment (*b* = 0.23, β = 0.27, *SE* = 0.08, *p* < 0.001), job satisfaction (*b* = 0.36, β = 0.38, *SE* = 0.08, *p* < 0.001), and work engagement (*b* = 0.35, β = 0.35, *SE* = 0.07, *p* < 0.001), supporting hypotheses 3a-e.

As Table 4 illustrates, the indirect effects of ethical leadership on the various follower outcomes were significant in each case, indicating that in support of hypotheses 4a-e, personal power mediated the relationship between ethical leadership and leader effectiveness (β = 0.48), follower extra effort (β = 0.66), organizational commitment (β = 0.16), job satisfaction (β = 0.26), and work engagement (β = 0.25). Significance tests for the indirect effects were based on bias-corrected confidence intervals derived from 10000 bootstrapped samples (Shrout and Bolger, 2002).

**Table 4:** Tests of indirect effects.

**Indirect paths**	**Indirect effect**	**Confidence interval**
Ethical leadership → personal power → leader effectiveness (H4a)	0.48 (0.06)	[0.37, 0.61]
Ethical leadership → personal power → follower extra effort (H4b)	0.66 (0.08)	[0.51, 0.83]
Ethical leadership → personal power → organizational commitment (H4c)	0.16 (0.05)	[0.07, 0.28]
Ethical leadership → personal power → job satisfaction (H4d)	0.26 (0.04)	[0.15, 0.39]
Ethical leadership → personal power → work engagement (H4e)	0.25 (0.06)	[0.15, 0.38]

*N = 235. Significance tests for the indirect effects were based on bias-corrected confidence intervals derived from 10,000 bootstrapped samples (Shrout and Bolger, 2002). Confidence level of confidence interval = 95%, standard errors are in parentheses*.

**Figure 2:**
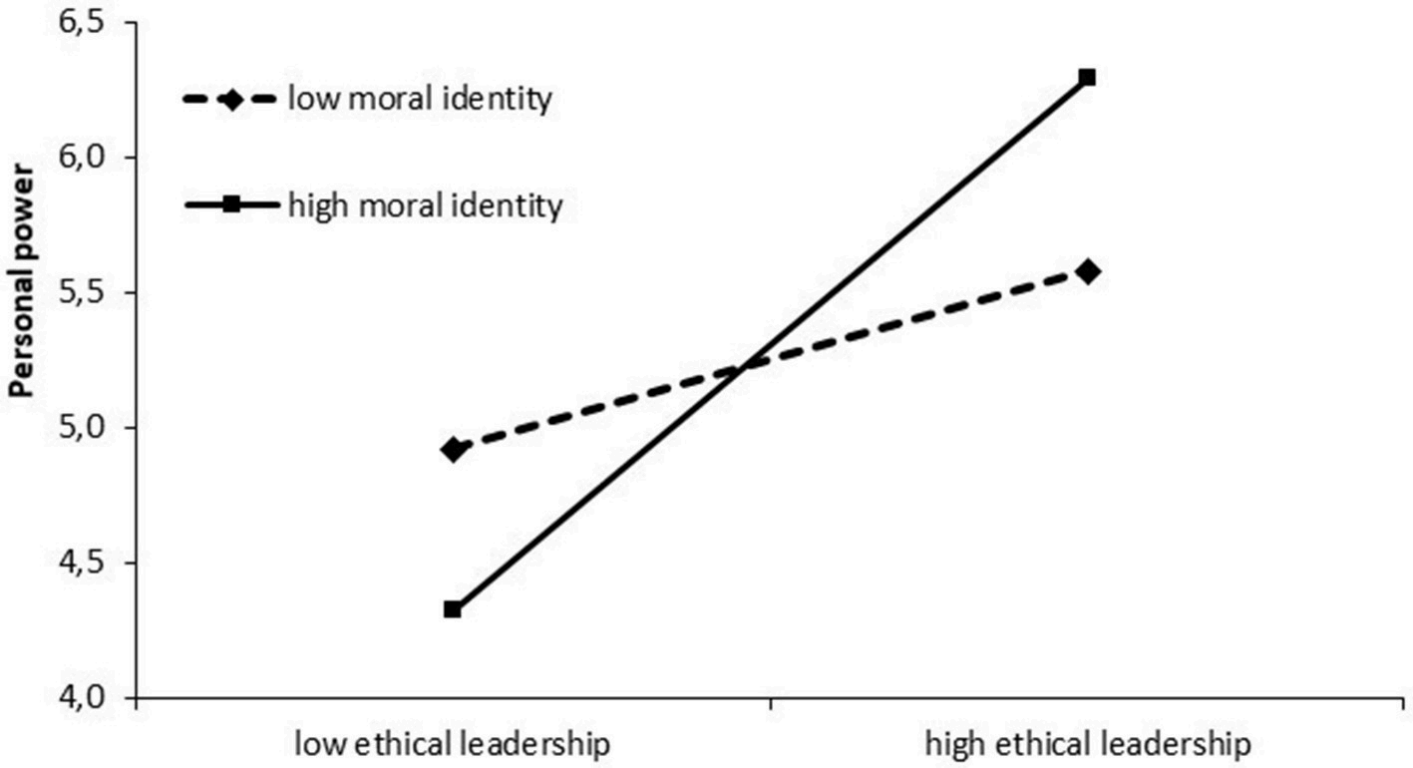
Moderating effect of an employee's moral identity on the relationship between ethical leadership and personal power.

Hypothesis 5 predicted that an employee's moral identity would moderate the relationship between ethical leadership and personal power. Table 2 reveals that the interaction term between ethical leadership and moral identity is positively related to personal power (*b* = 0.25, β = 0.18, *SE* = 0.08, *p* < 0.05). We plotted the interaction effect of ethical leadership and an employee's moral identity on personal power, as illustrated in Figure 2.

Moral identity moderated the relationship between the attribution of personal power bases and ethical leadership behavior, such that employees with a high moral identity attributed more personal power bases to their leader in case of high ethical leadership behavior and less personal power bases in case of low ethical leadership behavior compared to employees with low moral identity. Testing the conditional effect of ethical leadership on personal power on three values of moral identity (1 *SD* below the mean, the mean, 1 *SD* above the mean), simple slope analyses revealed solely significant effects (1 *SD* below the mean: slope: β = 0.46, *t* = 3.04, *p* < 0.01; the mean: slope: β = 0.66, *t* = 7.99, *p* < 0.001; 1 *SD* above the mean: slope: β = 0.86, *t* = 9.11, *p* < 0.001). Thus, hypothesis 5 is supported.

The results from the corresponding moderated mediation models show significant conditional indirect effects of ethical leadership on each follower outcome at the three tested values of moderator moral identity (see Table 5). Therefore, hypotheses 6a-e are supported, since personal power mediated the interactive effect of ethical leadership and an employee's moral identity on leader effectiveness (β = 0.32–0.59), follower extra effort (β = 0.43–0.80), organizational commitment (β = 0.11–0.20), job satisfaction (β = 0.17–0.31), and work engagement (β = 0.16–0.30).

**Table 5:** Tests of conditional indirect effects.

**Moderated mediation models*****: Ethical leadership x moral identity***→***personal power**→**follower outcome***										
**Follower Outcome**	**Leader effectiveness**		**Follower extra effort**		**Organizational commitment**		**Job satisfaction**		**Work engagement**	
**Level**	**Conditional indirect effect**	**CI**	**Conditional indirect effect**	**CI**	**Conditional indirect effect**	**CI**	**Conditional indirect effect**	**CI**	**Conditional indirect effect**	**CI**
Moral identity_low_	0.32 (0.10)	[0.13, 0.51]	0.43 (0.13)	[0.17, 0.69]	0.11 (0.05)	[0.03, 0.23]	0.17 (0.06)	[0.06, 0.31]	0.16 (0.06)	[0.06, 0.31]
Moral identity_mean_	0.45 (0.06)	[0.34, 0.59]	0.62 (0.08)	[0.46, 0.79]	0.15 (0.05)	[0.07, 0.27]	0.24 (0.06)	[0.14, 0.37]	0.23 (0.06)	[0.14, 0.36]
Moral identity_high_	0.59 (0.07)	[0.45, 0.74]	0.80 (0.10)	[0.60, 1.01]	0.20 (0.06)	[0.09, 0.33]	0.31 (0.07)	[0.18, 0.42]	0.30 (0.07)	[0.18, 0.44]

*N = 235. Moral identity was −0.82 (i.e., 1 SD below the mean) and.82 (i.e., 1 SD above the mean) for low and high levels of moral identity, respectively. Significance tests for the conditional indirect effects were based on bias-corrected confidence intervals derived from 10000 bootstrapped samples (Shrout and Bolger, 2002). Confidence level of confidence interval (CI) = 95%, standard errors are in parentheses*.

### Discussion study 1

In addition to positive relationships between ethical leadership and various follower outcomes (leader effectiveness, follower extra effort, organizational commitment, job satisfaction, and work engagement), which replicates the current state of research, the results of study 1 fully confirm our proposed process model. Thus, the attribution of personal power bases to a leader by his or her follower mediated the positive relationship between ethical leadership and each tested follower outcome, indicating a new power-based explanatory mechanism of the ethical leadership-follower outcomes link. Furthermore, the findings indicate the moderating role of an employee's moral identity in this process, such that the effect of ethical leadership on the perception of a leader's personal power was stronger for employees with high moral identities than for those with low moral identities, ultimately increasing follower outcomes, as personal power mediated the interactive effect of ethical leadership and an employee's moral identity on each follower outcome in the context of the moderated mediation models.

A limitation of study 1 represents the potential for common method variance, since the measures of every study variable stemmed from the same source (Podsakoff et al., 2003). This choice of data source is generally appropriate since all study variables intended to measure employees' attitudes, which cannot be captured by alternative sources. Leader effectiveness, as rated by followers, also represents a more valid behavior description than leader self-report (Kim and Yukl, 1995). Although findings indicate that the concern of common method bias in self-report data is overstated (Doty and Glick, 1998; Spector, 2006), we adopted three strategies to minimize common method bias. Hence, we collected the data at two measurement times (Podsakoff et al., 2003) and examined the factor structure of the measures, affirming the construct distinctiveness of all measured study variables. We also tested moderated mediation models, whose probability to be detected are seriously decreased in case of artificially inflated relationships (Edwards and Lambert, 2007). Thus, we are confident that common method variance played a minor role in our findings in the context of the (moderated) mediation models. Moreover, common methods variance leads to a 26% bias in the observed relationships among constructs (Doty and Glick, 1998). Assuming a reduction of 26% regarding the strength of the observed relationships, the effect sizes of the relevant regressors still correspond to at least a small effect, in most cases to medium or strong effects (Cohen, 1988). Thus, common method bias might not invalidate our findings.

Although exhibiting high external validity, the design of the field study does not allow causal conclusions about the hypothesized relationships. Thus, we conducted a second study; a scenario experiment in whose context ethical leadership (low ethical leader vs. high ethical leader) was manipulated to test our proposed power-based process model of the ethical leadership-follower outcomes link in a controlled laboratory setting.

## Study 2 (scenario experiment)

### Materials and methods

#### Procedure

The scenario experiment was implemented online, and participants were recruited on university-related social media platforms via postings. As an incentive, the participants could either receive course credits at the collaborating universities or register in a lottery. Participation in study 1 was defined as an exclusion criterion. The scenario experiment started with an introduction, in which participants were told that they would read a job-related scenario that focused on a leader's behavior. They were then asked to empathize with the employee described in the scenario and answer the subsequent questions according to their subjective estimates of the situation. The content of the short scenario described the work situation of a young professional in a reputable consulting firm. The manipulation of ethical leadership consisted of the team leader's description as either highly ethical or unethical, using van Gils et al.'s (2015) ethical leadership manipulation texts. After reading the scenarios, participants completed a series of questions regarding the manipulation check, rating the leader's personal power, and several follower outcomes predicted in response to dealing with the described work situation. Finally, the participants rated their own moral identity and provided their demographic data.

#### Sample

169 persons participated in the scenario experiment, who were randomly assigned to the conditions of a 2 factorial design [high ethical (*n* = 85) vs. low ethical leadership (*n* = 84)]; 70.4% of the sample was female and the average age was 25.3 years (*SD* = 5.62). Participants predominantly held a higher education entrance qualification (39%) or a university degree (58%; secondary modern school qualification or secondary school leaving certificate: 3%), and the overwhelming proportion of the sample (83.5%) was university students (1.8% unemployed; 0.6% housewife/househusband; 1.8% trainee; 11.2% employed; 1.2% self-employed).

#### Measures

Following van Gils et al. (2015), we used one item—“to what extent do you believe that your team leader is an ethical leader?”—as manipulation check of the scenario manipulations (1 = not ethical at all, 7 = very ethical). To measure the attribution of personal power bases, the participants' moral identity as well as the follower outcomes, we applied primarily the same scales as in the field study. Personal power was assessed with the Hinkin and Schriesheim's (1989) eight-item measure (α = 0.91); moral identity was measured with Aquino's and Reed's (2002) five-item scale (α = 0.81); follower extra effort (α = 0.92) and perceived leader effectiveness (α = 0.87) were evaluated with three and four items, respectively, of the MLQ (Bass and Avolio, 1995); work engagement (α = 0.96) was reported by the UWES-9 (Schaufeli et al., 2006), and organizational commitment was captured by Meyer et al.'s (1993) two subscales of affective commitment and normative commitment merged into one scale (α = 0.88). Contrarily, global job satisfaction was assessed with one item (“Altogether, how satisfied were you with your job at the consulting firm under the leadership of your supervisor?”), since a single item approach seemed more appropriate for the scenario's context, representing a valid and acceptable alternative for measuring overall job satisfaction (Wanous et al., 1997).

#### Control variables

Apart from controlling for the variables education level (1 = no school-leaving qualification to 7 = doctoral degree) and participants' sex (1 = “male,” 2 = “female”) for same reasons as in case of the field study, we controlled for two factors which might influence the quality of the questionnaire completion by affecting the amount of empathizing with the employee's role in the scenario. Thus, we controlled for the participants' perceived ability of their own imagination (“How hard was it for you to put you in the position of the employee?”; 1 = not hard at all, 7 = very hard) and their working experience as employees, measured on a six-point scale (1 = no experience to 6 = >5 years of experience as an employee).

#### Construct validity

Conducting a series of confirmatory factor analyses with AMOS (Brown, 2006), we examined the discriminant validity of our study variables based on chi-square statistics and fit indices of RMSEA, IFI, and CFI (Anderson and Gerbing, 1988; Joreskog, 1993). Following the procedure in study 1, we used the subscales in case of the constructs of personal power, work engagement, and organizational commitment. The hypothesized six-factor model of personal power, moral identity, leader effectiveness, follower extra effort, organizational commitment, and work engagement, χ^2^ (137, *N* = 169) = 254.90, *p* < 0.001; RSMEA = 0.07; IFI = 0.95 and CFI = 0.95, yielded a better fit to the data than a one-factor model, χ^2^ (152, *N* = 169) = 1025.72, *p* < 0.001; RSMEA = 0.19, IFI = 0.63, and CFI = 0.63, supporting the distinctiveness of the six study variables for subsequent analyses.

### Results

#### Manipulation check

An unrelated *t-*test with ethical leadership as the dependent variable confirmed that participants in the high ethical leadership condition perceived the described leader as significantly more ethical (*M* = 6.04, *SD* = 1.15) than participants in the low ethical leadership condition (*M* = 2.17, *SD* = 1.20; *t*_(167)_ = 21.40, *p* < 0.001). This result indicates that the manipulation was successful at construing a scenario with a highly ethical or an unethical leader.

#### Hypothesis testing

Table 6 reveals descriptive statistics and bivariate correlations of the study variables.

**Table 6:** Descriptive statistics and correlations.

	**Variable**	***M***	***SD***	**1**	**2**	**3**	**4**	**5**	**6**	**7**	**8**	**9**	**10**	**11**	**12**
1	Ethical leadership (manipulated)	0.50	0.50	1											
2	Moral identity	5.64	1.09	0.06	1										
3	Personal power	4.48	1.34	0.73^***^	0.05	1									
4	Leader effectiveness	4.20	1.39	0.72^***^	−0.04	0.82^***^	1								
5	Follower extra effort	4.44	1.46	0.51^***^	−0.08	0.70^***^	0.77^***^	1							
6	Org. commitment	3.86	1.03	0.53^***^	−0.02	0.60^***^	0.57^***^	0.55^***^	1						
7	Job satisfaction	4.49	1.71	0.65^***^	−0.06	0.73^***^	0.71^***^	0.57^***^	0.73^***^	1					
8	Work engagement	4.36	1.25	0.56^***^	0.07	0.66^***^	0.62^***^	0.58^***^	0.64^***^	0.77^***^	1				
9	Participant sex	1.70	0.46	0.06	0.18^*^	0.10	−0.01	0.06	0.10	−0.00	−0.04	1			
10	Participant education	4.67	0.81	0.08	0.14	0.00	0.07	−0.01	−0.06	0.01	0.03	0.16^*^	1		
11	Lack of imagination	3.41	1.44	−0.08	−0.19^*^	−0.05	−0.06	0.03	0.01	−0.06	−0.16^*^	0.02	−0.04	1	
12	Working experience	3.28	1.46	0.08	0.06	0.04	0.06	0.02	−0.02	0.02	0.07	−0.03	0.31^***^	−0.06	1

N = 169. For ethical leadership, 0 = low ethical leader, 1 = highly ethical leader. For participant sex, 1 = male, 2 = female. Org., organizational.

* p < 0.05;

**p < 0.01;

*** *p < 0.001*.

We applied the same procedures for hypothesis testing as in study 1. Manipulated ethical leadership was dummy-coded (0 = low ethical leadership, 1 = high ethical leadership). Therefore, to test hypotheses regarding the direct relationship between manipulated ethical leadership and follower outcomes (i.e., H1a-e), OLS regressions were estimated, controlling for participant's sex and education, lack of imagination during the scenario experiment, and working experience. The results show that ethical leadership predicted reported leader effectiveness (*b* = 2.00, β = 0.72, *SE* = 0.15, *p* < 0.001), follower extra effort (*b* = 1.52, β = 0.52, *SE* = 0.20, *p* < 0.001), organizational commitment (*b* = 1.11, β = 0.54, *SE* = 0.14, *p* < 0.001), job satisfaction (*b* = 2.23, β = 0.66, *SE* = 0.20, *p* < 0.001), and work engagement (*b* = 1.37, β = 0.55, *SE* = 0.16, *p* < 0.001), supporting hypotheses 1a-e.

As in study 1, hypothesis testing in the context of the mediation and moderated mediation models followed path analytic procedures (Edwards and Lambert, 2007; Preacher et al., 2007) and bootstrapping analyses to estimate the (conditional) indirect effects (Shrout and Bolger, 2002), applying the SPSS macro PROCESS (Preacher et al., 2007; Hayes, 2013). The two steps of the path analytic procedures are illustrated in Table 7 (regression results of the first-stage dependent variable of personal power) and Table 8 (regression results of the second-stage dependent variables; i.e., follower outcomes).

**Table 7:** First paths of the (moderated) mediation models, predicting the first stage dependent variable personal power.

**Models**	**Mediation Personal power**			**Moderated mediation Personal power**		
**Variable**	***b***	**β**	***SE***	***b***	**β**	***SE***
Ethical leadership	**2.00**	0.74^***^	**0.05**	2.00	0.74^***^	0.05
Moral identity				0.07	0.06	0.06
EL x moral identity				**0.47**	0.19^***^	**0.06**
Participant sex	0.21	0.07	0.06	0.12	0.04	0.06
Participant education	−0.11	−0.07	0.06	−0.11	−0.06	0.06
Lack of imagination	0.00	0.00	0.06	0.03	0.03	0.06
Working experience	0.00	0.00	0.05	−0.01	−0.01	0.05
*F*		38.89^***^			31.73^***^	
*R*^2^		0.55			0.58	

N = 169. For ethical leadership, 0 = low ethical leader, 1 = highly ethical leader. For participant sex, 1 = male, 2 = female. EL = Ethical leadership. Standard errors are based on standardized coefficients. Values in bold are relevant to hypothesis tests. *p < 0.05; **p < 0.01;

*** *p < 0.001*.

**Table 8:** Second paths of the (moderated) mediation models, predicting the second stage dependent variables, i.e., follower outcomes.

	**Leader effectiveness**			**Follower extra effort**			**Org. Commitment**			**Job satisfaction**			**Work engagement**		
**Variable**	***b***	**β**	***SE***	***b***	**β**	***SE***	***b***	**β**	***SE***	***b***	**β**	***SE***	***b***	**β**	***SE***
Ethical leadership	0.65	0.24^***^	0.07	0.04	0.01	0.08	0.47	0.23^*^	0.11	0.84	0.25^**^	0.09	0.32	0.13	0.10
Personal power	**0.68**	0.65^***^	**0.08**	**0.76**	0.69^***^	**0.09**	**0.33**	0.43^***^	**0.12**	**0.71**	0.56^***^	**0.09**	**0.54**	0.57^***^	**0.10**
Participant sex	−0.30	−0.10	0.05	−0.05	−0.01	0.06	0.12	0.05	0.07	−0.28	−0.07	0.06	−0.29	−0.11	0.06
Participant education	0.10	0.06	0.04	−0.02	−0.01	0.06	−0.10	−0.08	0.07	0.01	0.01	0.09	0.04	0.03	0.07
Lack of imagination	−0.01	−0.01	0.05	0.07	0.07	0.06	0.03	0.04	0.07	−0.01	−0.02	0.06	−0.11	−0.12	0.07
Working experience	−0.01	−0.01	0.04	−0.01	−0.01	0.06	0.05	−0.03	0.07	−0.03	−0.03	0.05	0.02	0.02	0.07
*F*		69.00^***^			21.93^***^			14.93^***^			32.88^***^			29.39^***^	
*R*^2^		0.71			0.49			0.39			0.57			0.48	

N = 169. For ethical leadership, 0 = low ethical leader, 1 = highly ethical leader. For participant sex, 1 = male, 2 = female. Org. = organizational. Standard errors are based on standardized coefficients. Values in bold are relevant to hypothesis tests.

* p < 0.05;

** p < 0.01;

*** *p < 0.001*.

The results from the mediation models revealed that ethical leadership was positively associated with personal power (*b* = 2.00, β = 0.74, *SE* = 0.05, *p* < 0.001; see Table 7), substantiating hypothesis 2. In addition, personal power was positively related to each follower outcome (see Table 8) including leader effectiveness (*b* = 0.68, β = 0.65, *SE* = 0.08, *p* < 0.001), follower extra effort (*b* = 0.76, β = 0.69, *SE* = 0.09, *p* < 0.001), organizational commitment (*b* = 0.33, β = 0.43, *SE* = 0.12, *p* < 0.001), job satisfaction (*b* = 0.71, β = 0.56, *SE* = 0.09, *p* < 0.001), and work engagement (*b* = 545, β = 0.57, *SE* = 0.10, *p* < 0.001), confirming hypotheses 3a-e.

As Table 9 demonstrates, the indirect effects of ethical leadership on follower outcomes were significant for all outcomes, confirming hypotheses 4a-e, as personal power mediated the positive relationship between ethical leadership and leader effectiveness (β = 1.33), follower extra effort (β = 1.48), organizational commitment (β = .64), job satisfaction (β = 1.39), and work engagement (β = 1.05). Significance tests for the indirect effects were based on bias-corrected confidence intervals derived from 10,000 bootstrapped samples (Shrout and Bolger, 2002).

**Table 9:** Tests of indirect effects.

**Indirect paths**	**Indirect effect**	**Confidence interval**
Ethical leadership → personal power → leader effectiveness (H4a)	1.33 (0.18)	[1.01, 1.71]
Ethical leadership → personal power → follower extra effort (H4b)	1.48 (0.21)	[1.11, 1.91]
Ethical leadership → personal power → organizational commitment (H4c)	0.64 (0.18)	[0.30, 0.99]
Ethical leadership → personal power → job satisfaction (H4d)	1.39 (0.23)	[0.96, 1.87]
Ethical leadership → personal power → work engagement (H4e)	1.05 (0.18)	[0.71, 1.40]

*N = 169. Significance tests for the indirect effects were based on bias-corrected confidence intervals derived from 10,000 bootstrapped samples (Shrout and Bolger, 2002). Confidence level of confidence interval = 95%, standard errors are in parentheses*.

**Figure 3:**
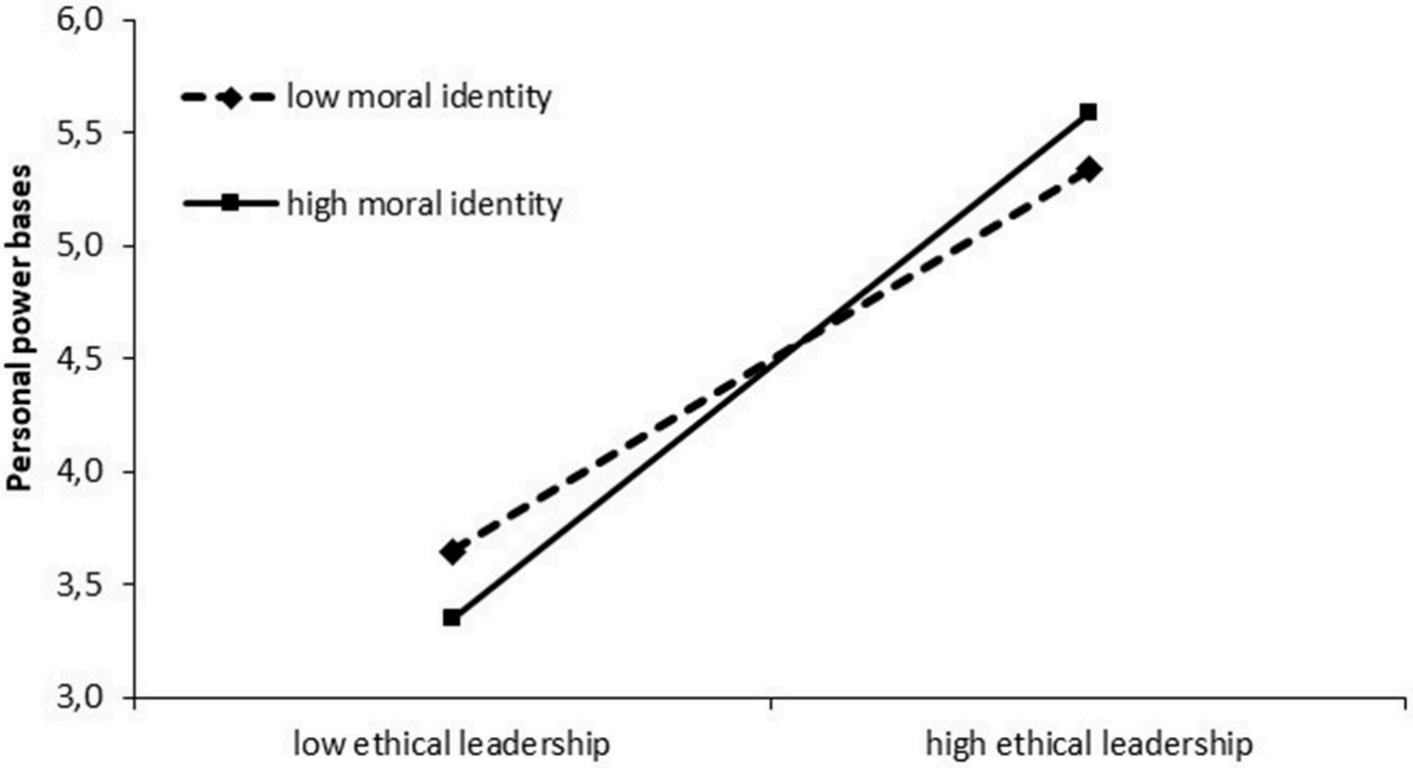
Moderating effect of a participant's moral identity on the relationship between ethical leadership and personal power.

Hypothesis 5 predicted that a participant's moral identity would moderate the relationship between ethical leadership and personal power. Table 7 reveals that the interaction term between ethical leadership and moral identity is positively related to personal power (*b* = 0.47, β = 0.19, *SE* = 0.06, *p* < 0.001). Plotting the interaction effect of ethical leadership and a participant's moral identity on personal power illustrates the moderating function of moral identity in the relationship between the attribution of personal power bases and ethical leadership in the scenario experiment, such that participants with a high moral identity attributed more personal power bases to their hypothetical leader in case of high ethical leadership behavior and perceived less personal power bases in case of low ethical leadership behavior, compared to participants with a low moral identity (see Figure 3).

Estimating the conditional effect of ethical leadership on personal power on three values of moral identity (1 *SD* below the mean, the mean, 1 *SD* above the mean), simple slope analyses showed solely significant effects (1 *SD* below the mean: slope: β = 1.44, *t* = 7.22, *p* < 0.001; the mean: slope: β = 1.96, *t* = 14.27, *p* < 0.001; 1 *SD* above the mean: slope: β = 2.47, *t* = 11.98, *p* < 0.001). Thus, hypothesis 5 is also supported by the scenario experiment's results.

Testing the corresponding moderated mediation models, analyses revealed significant conditional indirect effects of ethical leadership on each follower outcome at the three tested values of moderator moral identity (see Table 10). Therefore, hypotheses 6a-e are also substantiated by the scenario experiment, since personal power mediated the interactive effect of manipulated ethical leadership and a participant's moral identity on predicted leader effectiveness (β = 0.98–1.68), follower extra effort (β = 1.09–1.87), organizational commitment (β = 0.48–0.81), job satisfaction (β = 1.03–1.76), and work engagement (β = 0.77–1.33).

**Table 10:** Tests of conditional indirect effects.

	**Moderated mediation models*****: Ethical leadership x moral identity***→***personal power**→**follower outcome***									
**Follower Outcome**	**Leader effectiveness**		**Follower extra effort**		**Organizational commitment**		**Job satisfaction**		**Work engagement**	
**Level**	**Conditional indirect effect**	**CI**	**Conditional indirect effect**	**CI**	**Conditional indirect effect**	**CI**	**Conditional indirect effect**	**CI**	**Conditional indirect effect**	**CI**
Moral identity_low_	0.98 (0.17)	[0.68, 1.36]	1.09 (0.18)	[0.76, 1.50]	0.48 (0.13)	[0.24, 0.75]	1.03 (0.17)	[0.73, 1.44]	0.77 (0.14)	[0.53, 1.10]
Moral identity_mean_	1.33 (0.18)	[1.02, 1.72]	1.48 (0.21)	[1.11, 1.93]	0.64 (0.18)	[0.30, 0.99]	1.39 (0.23)	[0.96, 1.88]	1.05 (0.18)	[0.71, 1.41]
Moral identity_high_	1.68 (0.24)	[1.24, 2.18]	1.87 (0.29)	[1.34, 2.50]	0.81 (0.24)	[0.37, 1.29]	1.76 (0.34)	[1.13, 2.47]	1.33 (0.25)	[0.85, 1.84]

*N = 169. Moral identity was −1.09 (i.e., 1 SD below the mean) and 1.09 (i.e., 1 SD above the mean) for low and high levels of moral identity, respectively. Significance tests for the conditional indirect effects were based on bias-corrected confidence intervals derived from 10,000 bootstrapped samples (Shrout and Bolger, 2002). Confidence level of confidence interval (CI) = 95%, standard errors are in parentheses*.

### Discussion study 2

Based on the experimental manipulation of ethical leadership, the findings of study 2 replicate all the results of study 1, confirming the causal direction of ethical leadership's effect. The findings of the scenario experiment reveal that ethical leadership enhances both the attribution of personal power bases to leader and follower outcomes (i.e., leader effectiveness, follower extra effort, organizational commitment, job satisfaction, and work engagement). Furthermore, personal power bases mediated the positive relationship between ethical leadership and follower outcomes, elucidating ethical leadership's mechanism of action. In accordance with the field study results, the findings of study 2 indicate that an employee's moral identity moderates the process, such that a highly developed moral identity increases the positive effect of ethical leadership on the attribution of personal power bases to a leader, resulting in higher follower outcomes for participants with a high moral identity compared to those with a low moral identity.

Although the scenario experiment study design was suitable for testing the causal relationships implied in the proposed power-based process model, a coinciding limitation of this study relates to capturing reported hypothetical behavior as responses to a highly ethical or unethical leader. A scenario simulates a real-life setting, though the described situation may be experienced from an observer's perspective due to limited emotional empathizing with the illustrated circumstances, leading to diverging results compared to a field study (Kim and Jang, 2014). However, we likely confined that potential influence since we controlled for participants' experienced lack of imagination regarding their role in the described scenario. Future research could further substantiate our findings with a non-hypothetical experimental design in which participants step into a follower role in the context of a real leader-follower situation (see Damen et al., 2008).

## General discussion

By elucidating the transformation process of ethical leadership into follower outcomes from a power perspective, findings from both the two-wave field study (study 1) and the scenario experiment (study 2) confirm the proposed power-based process model of the ethical leadership-follower outcomes link. Thus, the attribution of personal power to a leader by his or her follower mediated positive relationships between ethical leadership and a broad range of follower outcomes, including leader effectiveness, follower extra effort, organizational commitment, job satisfaction, and work engagement. The results also reflected the moderating role of a follower's moral identity in this transformation process in terms of enhancing the relationship between ethical leadership and personal power. Accordingly, employees with a high moral identity attributed more personal power to their leader than employees with a rather low moral identity, subsequently resulting in higher follower outcomes, as personal power mediated the interactive effects of ethical leadership and a follower's moral identity on each tested follower outcome, such that the mediated effect was stronger when employees exhibited higher development levels of moral identity.

### Theoretical implications

The present study offers significant theoretical contributions to research on both ethical leadership and power.

Research on power bases is mainly limited to testing separately relations between power bases and leadership styles or rather follower outcomes (see Yukl, 2006; Rahim, 2009). Consistent with existing research that suggests personal power is closely connected to employee oriented leadership styles (e.g., Ansari, 1990; Pierro et al., 2013) and highly positively related to several beneficial follower outcomes (Yukl, 2006; Rahim, 2009), this study is one of the first to test power bases, more precisely personal power, as a mediating mechanism in a leadership behavior-follower outcomes link (see Pierro et al., 2013 for one exception). Thus, the present examination extends current state of research by first testing the direct relationship between power bases and an explicitly ethics related leadership style (i.e., ethical leadership), coincidently integrating two robust findings of the power research field into one influence chain. Furthermore, research on power bases has never examined the moderating influence of follower characteristics, such as moral identity, on the attribution of power bases. Thus, the present study offers a deepened insight in the leader behavior dependent process of attributing power bases by first explicating a conditional factor, namely follower personality (i.e., follower moral identity).

Research on ethical leadership has extensively explored the beneficial outcomes of ethical leadership behavior (Treviño and Brown, 2014) and has begun to examine the underlying mechanism of this correlation (e.g., Piccolo et al., 2010). However, the influencing process of ethical leadership on diverse follower outcomes has not been investigated from a power perspective, though the role of power, defined as the potency to influence (e.g., French and Raven, 1959), is highly relevant regarding the comprehension of an influence process. In this vein, the present study first examined the nature of influence in the context of ethical leadership in its purest form. Extending the current level of scientific knowledge on ethical leadership's mechanism of action, our findings consistently demonstrate a new power-based explanatory mechanism of the ethical leadership-follower outcomes link, as personal power mediated the positive relationship between ethical leadership and follower outcomes. On the basis of the attachment theory (Bowlby, 1969/1982), these results imply that first, ethical leadership behavior creates strong relational attachments, as the emotional bond between leader and follower is mirrored by the followers' attribution of personal power to a leader (see Bass, 2008) and second, that ethical leaders' effectiveness is partly based on their followers' perceptions of their leaders' personal power as highly developed. That means that the socially responsible power use in the context of ethical leadership describes a key explanatory mechanism in the ethical leadership-follower outcomes link. According to our findings, an ethical leader unfolds his or her influence on followers by personal appearance.

Moreover, the novel result of personal power as a mediator adds further insight in the theoretical foundations of the ethical leadership-follower outcomes link. In addition to indicating the attachment theory (Bowlby, 1969/1982) as plausible theoretical underpinning, the findings can be related to the social learning theory (Bandura, 1986), which serves as a common theoretical explanation for the relationship between ethical leadership and follower outcomes (Brown et al., 2005). The social learning theory implies that an ethical leader influences followers by role modeling (Brown et al., 2005). However, this proposition has been rarely examined. Two different studies showed that ethical leadership flows from one organizational level to the next (Mayer et al., 2009) and that leaders, who had ethical role models, tend to display more ethical leadership behavior (Brown and Treviño, 2014), indicating the function of role modeling in the ethical leadership process. Our findings indicate that personal power (i.e., the combination of expert and referent power) plays a critical intervening role in the relationship between ethical leadership and follower outcomes. As the core characteristic of referent power comprises perceiving a leader as a role model (Northouse, 2007), our finding leads to the conclusion that an ethical leader's influence on follower outcomes is indeed partly transferred by role modeling, as the attribution of referent power reflects the followers' perception of their leader as a role model (see Northouse, 2007). Thus, our findings offer one of the first empirical evidence that support the social learning theory (Bandura, 1986) as a significant theoretical underpinning of the link between ethical leadership and follower outcomes.

In addition to indicating a new explanatory mechanism of the ethical leadership-follower outcomes link, our findings imply a new defining element of the ethical leadership concept by capturing it from a power perspective. Power plays a key role in the leader-follower relationship (Yukl, 2006), and the socially responsible use of power is assumed as a key factor of ethical leadership (De Hoogh and Den Hartog, 2009). Our findings imply that typical ethical leadership behaviors convey high personal power, as the present examination offers the first-time empirical indication that ethical leadership is linked to (study 1), respectively creates (study 2) enhanced follower perceptions of a leader's personal power. Since personal power is considered as the most positive power source (e.g., Carson et al., 1993; Yukl, 2006), this finding indicates socially responsible power use within the framework of ethical leadership behavior.

Another theoretical implication concerns the moderating role of a follower's moral identity in the power-based transformation process of ethical leadership into follower outcomes. In both studies, a high follower moral identity enhanced the direct relationship between ethical leadership and a leader's personal power, leading to higher follower outcomes compared to a low moral identity. Thus, moral identity seems to influence the evaluation of ethical leadership behavior by affecting followers' perceptions of power messages transferred by ethical leadership behavior. Confirming the influence of the follower personality on ethical leadership's effect on follower outcomes is consistent with the current state of research, indicating that individual differences related to morality, such as moral emotions, increase the positive effect of ethical leadership on follower outcomes (see e.g., Eisenbeiss and van Knippenberg, 2015; van Gils et al., 2015). Extending the current research state, the present study first examined the moderating function of follower moral identity as a self-concept based personality variable, thus adding further insight in the followers' active role in the ethical leadership process. Coincidently, our findings contradict the results of several studies that detected the effects of ethical leadership on an employee's moral identity (Wen and Chen, 2016; Gerpott et al., 2017; Bavik et al., 2018), as ethical leadership and moral identity were unrelated in both of our studies, but they exhibited an interaction effect. Thus, the present studies add a new perspective on the relationship between ethical leadership and follower moral identity.

### Strengths, limitations, and future research directions

A prominent strength of our examination consists of combining an experimental design with a field study, since the strengths and weaknesses of these two methods simultaneously offset each other (see e.g., Damen et al., 2008). While granting high external validity, the two-wave single-source-field study does not permit causal conclusions and involves with the risk of common method bias. Conversely, the scenario experiment permits the exploration of causality, while lacking in realism due to the hypothetical context. The fact that the use of the two different methodologies allowed for the same conclusions increases confidence in the findings' robustness.

In addition to the limitations resulting from the study designs, a general limitation of our examination refers to the potential self-selection bias of web surveys (Bethlehem, 2010) that could confine the findings' generalizability. Although internet recruitment methods are approved by the American Psychological Association's Board of Scientific Affairs' Advisory Group (Kraut et al., 2004), future research could use probability sampling, for example by means of an appropriate online panel, to rule out the possibility that our conclusions only apply to a specific population (Bethlehem, 2010). In that respect, an investigation in an international context would be preferable to further extend the generalizability of results (Bond, 1998).

The present investigation offers options for future research to extend our findings.

One possible research direction concerns the examination of additional follower outcomes. As we exclusively investigated follower-rated attitudes, future research could also test the proposed power-based process model regarding behavior-based follower outcomes, including follower performance (Williams and Anderson, 1991) and organizational citizenship behavior (Lee and Allen, 2002). Since these variables are appropriate for using external ratings, the exploration of these behavioral follower outcomes could address the methodological issues of single-source data and extend the scope of the power-based process model.

A further option for future research refers to the expanded exploration of the influence chain implied in our model. To date, the explanatory mechanism of the link between the personal power and advantageous follower outcomes is mostly unexplored (Podsakoff and Schriesheim, 1985; Mossholder et al., 1998), and our model does not explain this relationship. Therefore, future research could integrate our finding on a leader's personal power as a mediator in the ethical leadership-follower outcomes link with study results on other diverse mediating mechanism, such as ethical climate (Neubert et al., 2009) or trust (Chughtai et al., 2015) to create a sequential mediation model, in which personal power functions as preceding mediator variable of another.

De Hoogh and Den Hartog (2009) argued that follower trust toward a leader may result from the socially responsible power use within the framework of ethical leadership behavior, thus mediating the positive relationship between ethical leadership and follower outcomes. Similarly, Neubert et al. (2009) theoretically deduced that ethical climate is a mediator of the ethical leadership-follower outcomes link by explaining an ethical leader's influence on ethical climate, among other factors, in terms of their personal power. Consistent with these theoretical considerations, Mossholder et al. (1998) showed that procedural justice mediated the relationship between a leader's personal power and followers' affective work reactions. Thus, it might be a fruitful approach to extend our model to a sequential mediation model by adding and testing further mediators that might arise from the attribution of personal power to a leader to further elucidate the personal-power-based process of transforming ethical leadership behavior into follower outcomes.

Finally, future research could examine the moderating role of environmental factors in the relationship between ethical leadership and the attribution of personal power bases. Since culture influences the perceptions of power bases (Aguinis et al., 1995, cited from Aguinis et al., 1998), it might be useful to examine the moderating role of organizational ethical culture (Treviño and Weaver, 2003). Considering the shaping function of an environmental factor could contribute to a more balanced perspective on the mechanism of the power-based transformation process of the ethical leadership-follower outcomes link.

### Practical implications

Our findings reveal several practical implications. The most obvious is based on the de novo empirical evidence confirming the organizational effectiveness of ethical leadership behavior. Thus, it might be useful for organizations to consider the ethical dimension of leadership behavior in leader selection and particularly leader development, as a well-designed leadership program can significantly promote ethical leadership behavior (van Velsor and Ascalon, 2008).

The findings also reveal that the effectiveness of ethical leadership is partly dependent on an employee's self-concept. This recognition could be implemented by means of employee selection. In particular, companies with organizational cultures that are highly characterized by an ethical dimension should pay attention to the development level of a potential employee's moral identity due to the expected increase in follower outcomes. In addition, a training approach may be a promising option since moral identity seems dependent on situational factors to some extent (e.g., Aquino et al., 2009).

A further managerial implication concerns the power aspect. Findings indicate that personal power plays an important role in the ethical leadership process and is the direct antecedent of various follower outcomes. This evidence highlights that power is an integral part of leadership and the responsible use of power can evolve highly advantageous outcomes for organizations, reducing the wide-spread fear of power, as the phenomenon of power and its function in organizations is still commonly denied in business contexts (Knoblach et al., 2012). Furthermore, the fact that ethical leadership behavior represents a good way to promote personal power is significant for managers. Regarding the current trend to flatter hierarchies (Erker et al., 2010), which involves the reduction of positional power, personal power as an incremental influence option may become the primary mechanism for exerting influence in organizational settings, and promoting one's own career advancement.

## Conclusion

By capturing an ethical leader's influence process on follower outcomes from a power perspective, our proposed power-based process model of the ethical leadership-follower outcomes link found substantial empirical support. We showed that a leader's personal power mediates the positive relationship between ethical leadership and various follower outcomes, suggesting that socially responsible power use within the framework of ethical leadership represents a key explanatory mechanism in the ethical leadership-follower outcomes link. Furthermore, our results confirm that a follower's moral identity plays a significant moderating role in this process by enhancing the positive effect of ethical leadership on a leader's personal power and subsequently on follower outcomes. Therefore, our findings concerning the influence process of ethical leadership on follower outcomes indicate that Baltasar Gracian's statement, “The sole advantage of power is that you can do more good,” builds the implicit guiding principle of an ethical leader.

## Data availability statement

Datasets are available on request: the raw data supporting the conclusions of this manuscript will be made available by the authors, without undue reservation, to any qualified researcher.

## Ethics statement

This study was carried out in accordance with the recommendations of the ethics committee of the Faculty for psychology and pedagogics, LMU Munich (http://www.fak11.lmu.de/forschung/ethikkommission/) with written informed consent from all subjects.

## Author contributions

DH, PF, and DF contributed to conception and design of the studies. DH collected the data, performed the statistical analysis, and wrote the manuscript. All authors contributed to manuscript revision, read and approved the submitted version.

### Conflict of interest statement

The authors declare that the research was conducted in the absence of any commercial or financial relationships that could be construed as a potential conflict of interest.

## Footnotes

^1^An unpublished study with another main research focus that surveyed employees (*N* = 225) showed that ethical leadership (Kalshoven et al., 2011) is unrelated to followers' perception of a leader's positional power (*r* = 0.09, *p* > 0.05) and highly related to perceptions of a leader's personal power (*r* = 0.81; *p* < 0.001).
